# Inflammatory Fibroid Polyp in a 48-Year-Old Male: A Rare Cause of Intussusception

**DOI:** 10.1155/2020/9251042

**Published:** 2020-01-03

**Authors:** Theresia Karuhanga, Caroline Ngimba, James J. Yahaya

**Affiliations:** ^1^Department of Surgery, Faculty of Medicine, St Francis Referral and Teaching Hospital, Ifakara, Morogoro, Tanzania; ^2^Department of Pathology, Mitokiti Histopathology Diagnostic Center, Dar es Salaam, Tanzania; ^3^Department of Pathology, Makerere University, School of Biomedical Sciences, Kampala, Uganda; ^4^Department of Biomedical Science, College of Health Sciences (CHS), University of Dodoma, Dodoma, Tanzania

## Abstract

Inflammatory fibroid polyp is a neoplastic condition affecting the gastrointestinal tract and particularly the gastric antrum. It is virtually a benign submucosal mass comprising mesenchymal cells and numerous small blood vessels with inflammatory cells and commonly eosinophils. Patients with inflammatory fibroid polyps usually present clinically with mechanical intestinal obstruction with or without intussusception. Herein, we present a case of a 48-year-old male with a known history of schizophrenia who presented with mechanical intestinal obstruction following intussusception due to inflammatory fibroid polyp involving the proximal jejunojejunal part of the jejunum.

## 1. Introduction

Inflammatory fibroid poly (IFP) is a rare benign solitary lesion that arises from the submucosa of the gastrointestinal tract (GIT) [1]. In 1949, Vaněk [2] first described the lesion as an eosinophilic submucosal granuloma. The occurrence of IFP along the GIT is as follows: 66-75% of the cases occur in the gastric antrum; 18-20% of the cases occur in the small intestine; 4-7% of the cases occur in the large intestine; 1% occurs in the duodenum, esophagus, and gallbladder; and less than 1% involves the appendix [2, 3]. However, the ileal segment is the most common site where these polyps cause intussusception [4]. IFPs can be found in all age groups, but peak incidence is between the sixth and seventh decades with a slight preponderance in males [1–3]. The estimated incidence of IFP in the general population is 0.3 to 0.5% [5].

The pathogenesis of IFP is still not clear. Studies recently have reported a possible sharing of the PDGFRA mutational profile with gastrointestinal stromal tumour (GIST) particularly exons 12 and 18 [6, 7]. This finding still shows contradicting results in the sense that other studies have reported that some IFP cases lack PDGFRA mutation [8, 9]. Although most of the cases are sporadic, a familial relationship has also been described [10]. The clinical presentation of patients with IFP is diverse. Symptoms and signs may vary depending on the GIT area involved [11]. Involvement of the antrum is often accompanied with vomiting, epigastralgia, and bleeding whereas involvement of the small bowel is associated with mechanical obstruction, particularly due to intussusception. Additionally, when IFP involves the large bowel, patients tend to present clinically with colicky pain, weight loss, diarrhea, bleeding, and anemia [3].

We report this case in order to provide source of reference in the literature as well as to inform the clinicians on the need of ruling out IFP cases by means of immunohistochemical (IHC) stains in order to rule out tumours that are commonly affecting the GIT such as GIST.

## 2. Case Presentation

A 48-year-old male with a known history of schizophrenia was brought at the Emergency Department (ED) of the referral and teaching hospital with a two-week history of abdominal pain, loss of appetite, and bilious vomiting; however, there was no history of abdominal distension or upper gastrointestinal bleeding. On physical examination, the patient was dehydrated and wasted with scaphoid abdomen. No obvious gastric distension was noticed, but there was positive succussion splash and visible epigastric peristaltic movements without an obvious palpable mass. The vital signs were as follows: BP = 86/60 mmHg, pulse rate = 75 beats/minute, and body temperature = 36.7°C. The provisional diagnosis was proximal small bowel obstruction due to bilious vomiting with differential of gastric outlet obstruction as a result of the presence of succussion splash and visible epigastric peristaltic movements.

**Figure 1:**
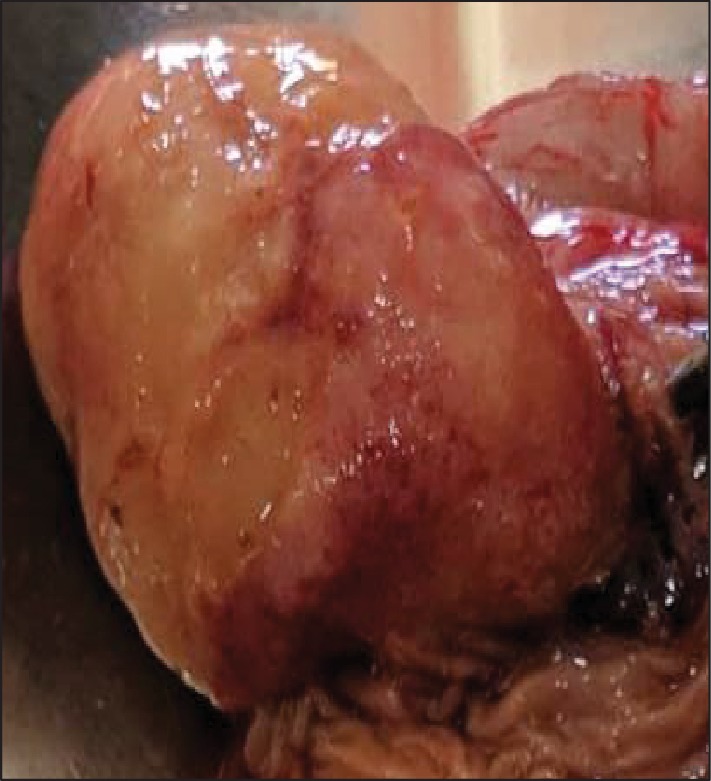
Macroscopic appearance of the intraluminal mass. The mass is submucosal, pedunculated, and ovoid, and there is intact overlying mucosal surface.

Resuscitation of the patient was done using normal saline intravenous (IV) fluids of 6000 mL for 12 hours for the purpose of restoring the hemodynamics. At the end of the 12 hours following fluid infusion, the BP turned to normal (110/74 mmHg). Then, a maintenance volume of 4500 mL of the fluid was added for another 6 hours. Initially, the stool was solid and later the stool turned watery. However, bilious vomiting persisted. Abdominal ultrasound scan was normal. Likewise, the plain abdominal X-ray was diagnostically nonspecific. On the 3rd day post admission, he underwent explorative laparotomy. Intraoperatively, a jejunojejunal intussusception was noticed at 18 cm from the duodenojejunal junction with intraluminal obstructing mass. On macroscopic examination, the mass was polypoid in shape and it was pedunculated. The size of the lesion was 4 × 3 × 3 cm, soft in consistency, and pinkish in appearance. Its lining mucosal surface was not ulcerated (Figure 1). There were no enlarged mesenteric lymph nodes that were detected. The intussuscepted loops were resected followed by direct end-to-end anastomosis.

**Figure 2:**
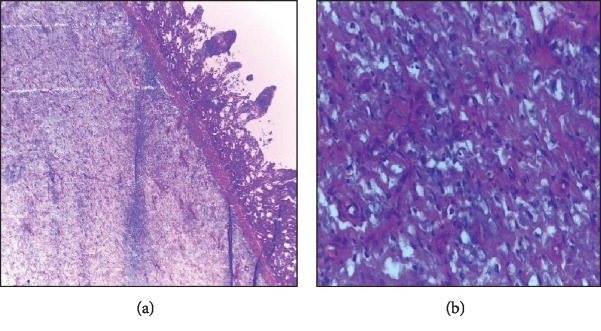
(a) Proliferating bland spindle tumour cells in the submucosa. The tumour shows bland short spindle cells haphazardly arranged in an edematous stroma (H&E, ×40). (b) Myxomatous stroma. There are sparse inflammatory cells in a myxomatous stroma with marked edema (H&E, ×40).

On microscopic examination, the tumour was found in the submucosa with blunting and edematous villi and it was extending to involve the muscularis propria with clearly defined margins. The tumour cells were spindle with bland nuclei and scanty eosinophilic cytoplasm (Figure 2(a)). Some areas of the lesion showed collagenized stroma, proliferation of medium-sized blood vessels, and infiltration of inflammatory cells mainly consisting of eosinophils in the background of myxomatous stroma (Figure 2(b)).

**Figure 3:**
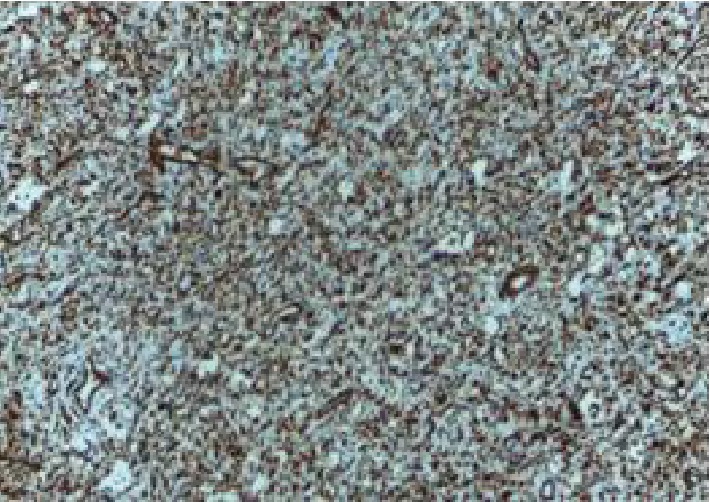
Strong and diffuse staining of the stellate cells. The spindle stromal tumour cells were not taking up the antibody compared to the stellate cell (IHC, ×40).

Immunohistochemical (IHC) staining for vimentin was strongly and diffusely positive (Figure 3) However, CD117 antibody was negative. This helped in ruling out the gastrointestinal stromal tumour (GIST). There was no postoperative complication. The patient was discharged five days after surgery. The follow-up period of five months was uneventful.

## 3. Discussion

The term IFP was first proposed by Helwig and Ranier [12] in 1953 for gastric polyps; since then, it has gained acceptance for similar lesions throughout the GIT. A hundred reports, mainly involving case series and reports due to lack of large data because of rarity nature of the lesion, are available in the literature, and most of them mainly focus on clinical and morphologic aspects. For a couple of decades since this condition was included in the literature, IFPs have been considered reactive changes of the lining mucosa of the GIT. The discovery of PDGFRA mutation signatures in IFPs has helped to unveil their neoplastic nature [13].

IFPs are always asymptomatic, and they remain silent for a long time without being diagnosed. Incidentally, they may be diagnosed during laparoscopic or endoscopic examination due to other underlying gastrointestinal (GI) medical conditions. As they become clinically detectable, the signs and symptoms usually depend on two factors: location and size of the tumour [14]. Studies have shown that IFPs that involve stomach; patients commonly present with abdominal pain, whereas those with small bowel involvement often present with intussusception and obstruction as it was in our case [15]. Intussusception caused by IFPs seem to have been reported by quite many authors.  Table 1 shows some of the cases of intussusception caused by IFP that have been reported in the literature.

Moreover, vomiting, diarrhea, tenesmus, and alterations in bowel habits are also seen although their frequencies are low. Larger polyps tend to erode and ulcerate superficially and lead to bloody stool. Patients with IFPs in the small bowel are most likely to present with chronic episodes of colicky abdominal pain, lower GI bleeding, anemia, and, more rarely, intestinal obstruction due to episodes of intestinal intussusception and rarely with necrosis and perforation [18]. Although malignant diseases represent the major causes of intussusceptions in adults, there are few reports of intestinal obstruction and perforation caused by IFPs. Preoperative diagnosis of intussusceptions is rare but can occur in finding a palpable mass on the abdomen with the use of imaging techniques.

The aetiopathogenesis of IFPs is unknown. Hypotheses have been postulated in the attempt to explain the cause of IFPs such as foreign body, parasites, and chronic *H. pylori* infection which have been suggested but remain unsupported [19]. Localized variant of eosinophilic gastroenteritis is another proposed aetiology due to frequent observation of a marked eosinophilic infiltration in most cases [13]. It was not possible to ascertain the predisposing factors that were associated with the development of this jejunojejunal IFP in our case. There is no previous report of intussusception arising from IFP at our institution and even the country at large. Our case indicates that this condition should be considered in elderly patients with features of chronic abdominal pain, vomiting, and frequent distension of the abdomen.

Abdominal computed tomography is currently considered the most sensitive imaging diagnostic technique to show the polyp or to confirm intussusceptions [19]. Most IFPs can be resected endoscopically. Only rarely is surgery necessary. IFPs arising below the Treitz ligament can present with an acute abdomen usually due to intussusceptions. Exploratory laparoscopy or laparotomy is frequently recommended as the best treatment for intussusceptions caused by IFP. The operation should be performed as early as possible in order to prevent the intussusceptions from leading to ischemia, necrosis, and subsequent perforation of the invaginated bowel segment [20].

Confirmation of IFPs at diagnosis requires the use of IHC stainings. This is because IFPs tend to resemble morphologically other mesenchymal tumours such as GIST, desmoid fibromatosis, inflammatory myofibroblastic tumour (IMT), leiomyoma, and schwannoma among others which are the histopathological differentials of IFPs. Most IFPs are positive for CD34, whereas about 10%–20% of cases show focal reactivity for SMA and desmin. C-KIT (CD117), DOG1, and S-100 are consistently negative [21].

Although IFPs are biologically regarded benign and noninvasive lesions [22], few cases have been observed to invade the muscularis propria layer [23]. With regard to gastric IFPs, only three invasive cases have been reported in the literature described in 2015 [24, 25]. Here, we describe a case of an invasive IFP involving the small bowel. In the present case, spindle tumour cells invaded the muscularis propria layer but had not invaded the subserosal layer. We believe that this is the first case in the literature describing invasive IFP of the small bowel. These findings imply that IFPs might occasionally behave as locally aggressive neoplasms with infiltrative growth patterns and may exhibit local recurrence after inadequate resection. Although endoscopic treatment is typically indicated for IFPs [24], when a lesion invades the muscularis propria layer, this treatment strategy can be inadequate. Indeed, a case of local recurrence after endoscopic removal has been reported [4].

## 4. Conclusion

Inflammatory fibroid polyps should be considered as one of the causes of intestinal obstruction due to intussusception among the polypoid masses affecting the GIT. Immunohistochemical staining panel comprising of C-KIT, CD34, SMA, and S-100 among many others ought to be deemed for differentiating IFPs from other mesenchymal tumours particularly GISTs. Use of abdominal CT scan is invaluable and should also be used in assessment of the patients with intestinal obstruction for detection of IFPs. Also, precaution must be taken not to leave behind the IFP masses in the patients due to chances of relapse even though the chances are said to be low.

## Figures and Tables

**Table 1:** Some previous IFP cases reported with intestinal obstruction due to intussusception.

Author(s)	Age (years)/sex	GI location	Greatest diameter (cm)
Bae et al. [8]	48/F	Ileum	3.5
Bjerkehagen et al. [6]	34/F	Ileum	3.8
Bjerkehagen et al. [6]	41/M	Ileojejunal	1.4
Jabar et al. [5]	34/M	Caecum	3.0
Jabar et al. [5]	47/M	Pylorus	2.3
Paikos et al. [16]	65/F	Antrum	5.0
Talukder et al. [3]	62/F	Jejunum	2.7
Jan et al. [1]	60/F	Ileum	4.0
Lasota et al. [7]	60/M	Ileocecal	4.6
Akbulut [17]	38/F	Ileum	3.8
